# Expression profiles of miR-101-3p and miR-431-5p as potential diagnostic biomarkers for rheumatoid arthritis

**DOI:** 10.1038/s41598-024-82339-1

**Published:** 2025-01-04

**Authors:** Mohamed M. Sadaty, Salma M. Mekhemer, Shaimaa Abdel-Ghany, Amira R. El-Ansary, Rana Mohamed, Nashaat N. Kamal, Hussein Sabit

**Affiliations:** 1 Department of Technology of Medical Laboratory, Faculty of Applied Health Science Technology, Misr University for Science and Technology, P. O. Box 77, Giza, Egypt; 2 Department of Environmental Biotechnology, College of Biotechnology, Misr University for Science and Technology, P. O. Box 77, Giza, Egypt; 3 Department of Internal Medicine, Faculty of Medicine, Misr University for Science and Technology, P. O. Box 77, Giza, Egypt; 4 Department of Medical Biotechnology, College of Biotechnology, Misr University for Science and Technology, P. O. Box 77, Giza, Egypt

**Keywords:** Rheumatoid arthritis (RA), Diagnostic biomarkers, miR-99b-5p, miR-101-3p, miR-431-5p, miRNAs, Molecular medicine

## Abstract

Rheumatoid arthritis (RA) is a chronic autoimmune disease characterized by persistent inflammation of the synovial joints, leading to cartilage and bone destruction. This study aimed to evaluate the diagnostic utility of specific microRNAs (miRNAs) as potential biomarkers for RA. The study was conducted on 60 patients with RA disease along with 20 control participants. Comprehensive analysis of patient data, encompassing serological, hematological, and biochemical markers, revealed significantly elevated levels of miR-99b-5p, miR-101-3p, and miR-431-5p in RA patients compared to healthy controls. Among these, miR-101-3p demonstrated the highest diagnostic accuracy, with an area under the curve (AUC) of 0.873. These findings contribute to a deeper understanding of RA pathogenesis and suggest that miR-101-3p may serve as a valuable biomarker for early disease detection and potentially improved patient management. Further research is warranted to elucidate the precise mechanisms underlying miRNA involvement in RA and to explore their potential as therapeutic targets.

## Introduction

Rheumatoid arthritis (RA) is one of the most prevalent autoimmune diseases, affecting around 1% of the population worldwide. RA is a chronic inflammatory sickness involving joints, leading to erosive synovitis, degradation of cartilage, and destruction of the joint. Long-term outcomes are indicated by joint structural damage, which contributes to functional failure, loss of ability and the need for major surgeries over time. RA is most common in the wrist and hands, with involvement of the same joints usually on both body sides^1^.

Arthralgia, edema, redness and even a reduction in range of motion are all symptoms of RA. Early diagnosis is regarded as a key improvement index for the most desirable outcomes (i.e., reduced joint destruction, less radiologic progression, no functional disability, and DMARD-free remission) as well as cost-effectiveness, as the first 12 weeks after the onset of early symptoms are considered the optimal therapeutic window^2^.

Several epidemiological studies of RA have been published in recent years, revealing differences in the incidence and prevalence of RA across populations. Most of the investigations were conducted in North European and North American countries, with prevalence rates ranging from 0.5 to 1.1%. The lowest incidence rates have been observed in Africa and Asia, while the greatest rates have been documented among Native American communities^3^.

Many epigenetic pathways that contribute to the pathophysiology of autoimmune illnesses have been discovered in the previous decade. Alterations in gene expression regulation through both microRNAs and long non-coding RNAs have been proposed to contribute to the pathogenesis of RA. microRNAs (miRNA) are non-coding RNA molecules with 18–24 nucleotides that regulate gene expression and have a key function in a variety of biological processes like cell differentiation and homeostasis by attaching to complementary sequences on messenger RNA (mRNA) and preventing target genes from being transcribed^4^.

Autoimmune diseases involve a complex dysregulation of immunity, and they reflect the interplay between environment and genetic factors. Autoimmune diseases include many members [e.g., RA and systemic lupus erythematosus (SLE)], and most of them are classified according to what organs and tissues are targeted by the damaging immune response^5^.

A series of miRNAs were found to be uncontrolled in subsets of cells inside the articular compartment of RA patients, leading to the production of proinflammatory cytokines and the activation of leukocytes, which take part in the immunologic component of effector synovial pathology. Numerous miRNAs related to inflammatory cytokines, synovial cell proliferation and osteoclast differentiation have been identified to date, and attempts have been made to use them in RA treatment^6^.

miR-431-5p has been implicated in the regulation of oxidative stress and apoptosis in various inflammatory conditions. Given the role of oxidative stress in RA pathogenesis, we hypothesize that miR-431-5p may affect cellular stress responses and contribute to the tissue damage observed in RA patients^7^.

Recent research has highlighted the potential of miRNAs as diagnostic biomarkers for RA. Due to their ability to regulate gene expression and immune responses, miRNAs can reflect pathological changes in RA at the molecular level. Specific miRNAs have been associated with disease activity and severity, suggesting their utility in not only diagnosing RA but also in monitoring disease progression. This makes miRNAs valuable tools for developing personalized treatment plans and improving patient outcomes^8,9^.

In the future, the role of miRNAs in RA diagnosis and management is expected to expand significantly. Advances in miRNA profiling technologies could lead to the development of robust, non-invasive diagnostic tests, allowing for earlier and more accurate detection of RA. Additionally, miRNA-based therapies are being explored as potential treatments to modulate immune responses and alleviate inflammation in RA patients. By integrating miRNA analysis into clinical practice, healthcare providers can achieve better disease management and improve the quality of life for individuals with RA^10,11^.

Differential expressions of circulating miR-99b-5p in the plasma of ERA patients may characterize a severe form of the disease. miR-99b-5p may serve as a possible predictor for erosion progression^12^.

miR-101-3p has been associated with the regulation of synovial fibroblast activation in RA, a key factor in joint destruction and inflammation. Research has demonstrated that miR-101-3p can inhibit the proliferation of synovial fibroblasts, thus reducing inflammatory responses and tissue damage^13^.

## Materials and methods

This study was conducted during the period from May 2022 to May 2023. Blood samples were collected from 60 RA patients (55 females and 5 males) attending to rheumatology unit in the Memorial Souad Kafafi University Hospital with age range (20–60 years). Clinical diagnosis of patients was confirmed by rheumatology specialists based on clinical features and serological, immunological & Hematological study (RF, CRP, ESR, ANA and Anti-CCP). Patients who included in this study were diagnosed with RA according to the ACR (American College of Rheumatology) classification criteria^14^.

### Data collection

Assessment sheet consisted of 3 parts:

Socio demographic data: Patient name (code), age, gender, residence, body mass index, occupation, and smoking habit.

Medical history: Family history, presenting symptoms, disease duration, number of tender and swollen joints, current treatment of RA and presence of other medical conditions.

### Biochemical, immunological, hematological, and molecular analysis

#### Blood sample collection

Venous blood samples (5 mL) were collected from RA patients attending the rheumatology unit, in the Memorial Souad Kafafi University Hospital with age range (20–60) years old. Also, venous blood samples (5 mL) were collected from the control group. A portion of blood sample was submitted to perform complete blood count, erythrocyte sedimentation rate and HbA1c immediately after obtaining of the samples, then stored at -80° C until miRNA extraction. Another portion of blood (3 mL) was admitted to clot and centrifuged at 4000 *xg* for 10 min. to separate serum used for assessment of SGPT, Creatinine, lipid profile, Anti-CCP, ANA, CRP, and RF. The research protocol was approved by the ethical guidelines of Misr University for Science and Technology’s Ethics Committee (FWA00025577). Informed consent was obtained from all subjects and/or their legal guardian(s). We confirm that all experiments were performed in accordance with the guidelines and regulations.

#### Determination of immunological and serological tests

Determination of anti-cyclic citrullinated peptide level, Anti-CCP kit supplied by ORGENTEC Diagnostika GmbH. The determination is based on an indirect enzyme linked immune reaction. Determination of antinuclear antibody level, ANA kit supplied by ORGENTEC Diagnostika GmbH. The determination is based on an indirect enzyme linked immune reaction. Determination of C- Reactive Protein level rheumatoid factor level turbidimetrically using fixed-time measurement.

#### Determination of hematological tests

Complete blood count test automatically by Sysmex instrument, Japan. The erythrocyte sedimentation rate (ESR) expresses in mm per hour, it is measured by the height of the column of clear plasma at the end of one hour.

#### Determination of biochemical tests

Determination of ALT, Creatinine, Lipid profile (Cholesterol-Triglycerides-HDL-LDL), Calcium, Phosphorous, Uric acid and HbA1c. Automatically by Erba Mannheim XL-180, Germany.

#### Calculating disease activity score (DAS28)

DAS28 is a measure of disease activity in RA (RA). DAS stands for ‘disease activity score’, and the number 28 refers to the 28 joints that are examined in this assessment. DAS28 is calculated according to the formula that is composed of the number of tender joints and swollen joints, and erythrocyte sedimentation rate (ESR).

#### Determination of micro-ribonucleic acids expression level

The expression level of circulating miRNAs (miR-431-5p, miR-101-3p, miR-99b-5p) were determined by two steps reverse transcription polymerase chain reaction (RT-PCR) technique.

#### Extraction of miRNA

Total ribonucleic acid (RNA), including miRNA was extracted from all EDTA blood samples of patients and controls using QIAzol^®^ lysis reagent supplied by QIAGEN, Germany (cat. no.339340).

#### Reverse transcription of micro-ribonucleic acids

The extracted miRNA of each sample was converted into cDNA with high specificity in a thermal cycler using microRNA reverse transcription kit and reverse transcription primers specific for each miRNA supplied by QIAGEN, Germany.

#### Quantitative real-time PCR

Quantitative real-time PCR (qPCR) was done using SYBR^®^ Green MicroRNA Assays which contain primer sequences specific for each miRNA.

The following procedures were used to perform qPCR: Initial heat activation 2 min. at 95° C, denaturation for 10 s. at 95° C, and combined annealing/extension 1 min. at 56° C.

#### Analysis of real-time PCR

The Ct value of target miRNA was normalized to an endogenous control or reference miRNA (hsa-miR-103a-3p) and relative to a healthy control. The expression of miRNAs was determined by relative quantitative method in which the fold change in the expression of patient’s miRNAs to the healthy control was calculated using the comparative Ct formula of ∆∆Ct).

### Statistical analysis

Using SPSS (Statistical Package for Social Science) program for statistical analysis, (version 26; Inc., Chicago. IL). Descriptive data were expressed as mean µ and standard deviation (SD). The student t-test was used to compare mean and SD of 2 sets of quantitative normally distributed data, one way analysis of Variance (ANOVA) test was used for comparison between three or more groups having quantitative normally distributed data. And significant differences were detected in P – value. 95 confidence intervals (CI) were calculated to evaluate the relationship between the different cases. Spearman & Pearson correlation coefficient for non-parametric & parametric values. The ROC (Receiver Operating Characteristic) curve was done to detect the cutoff value with highest sensitivity and specificity. P-value was considered statistically significant when it is less than 0.05.

## Results

Demographic data for different groups involved in the study were classified to 2 groups. Group A represents 60 patients having RA (5 Males (8.4%) & 55 Females (91.6%) with the mean value of their age was (43.2 ± 9.94), Group B represents 20 normal adults (control) (8 Males (40%) & 12 Females (60%), with the mean value of their age was (40.2 ± 7.66). According to family history, 52% of cases have a family history of RA and (48%) of cases have no family history. According to duration of disease, 50% of cases having RA since 1 to 5 years, 25% of cases having RA since 5 to 10 years, and 25% of cases having RA for more than 10 years. According to type of treatment, 55% of cases were under conventional treatment and 45% of cases under biological treatment. According to calculate DAS number results, RA cases divided to four subgroups 13.3% less than 2.6, 6.7% from 2.6 to 3.19, 46,7% from 3.2 to 5.1, 33.3% greater than 5.1 (Table 1).

**Table 1:** Demographic data for all participants.

Parameters	RA (*N* = 60)	Control (*N* = 20)
Age	43.2 ± 9.94	40.2 ± 7.66
Sex	5 Males (8.4%) 55 Females (91.6%)	8 Males (40%) 12 Females (60%)
Family history for RA cases		
Cases with positive family history		31 (52%)
Cases with negative family history		29 (48%)
Duration of the disease for RA cases		
Cases suffering from RA since 1 to 5 years		30 (50%)
Cases suffering from RA since 5 to 10 years		15 (25%)
Cases suffering from RA for more than 10 years		15 (25%)
Types of treatment for RA cases		
Cases under conventional DMARDs treatment		33 (55%)
Cases under biological DMARDs treatment		27 (45%)
DAS number of RA cases		
Less than 2.6 (Remission)		8 (13.3%)
From 2.6 to 3.19 (Low)		4 (6.7%)
From 3.2 to 5.1 (Moderate)		28 (46.7%)
Greater than 5.1 (High)		20 (33.3%)

**Fig. 1:**
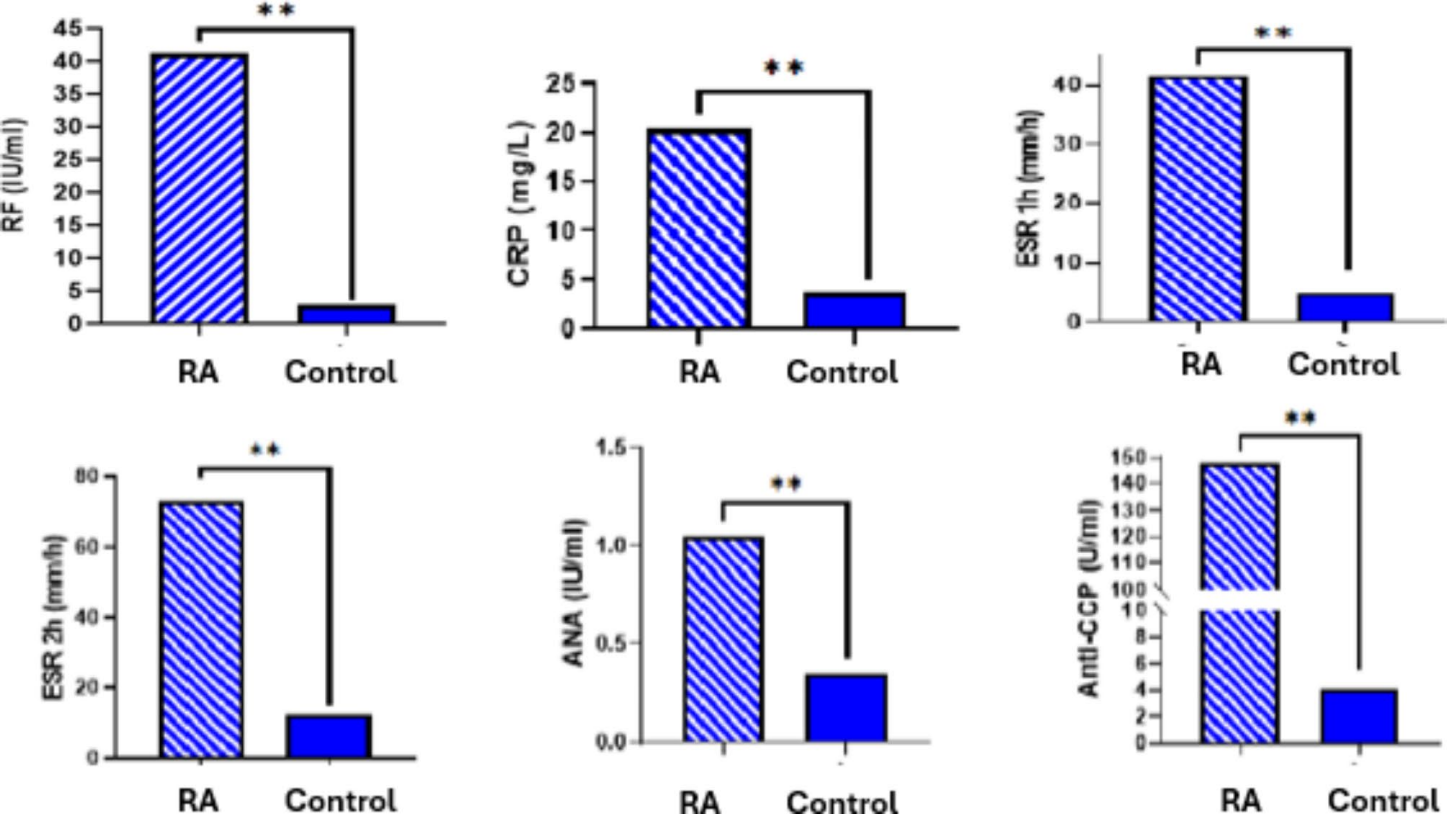
Immunological and hematological parameters for participants with RA compared to controls. The charts show significantly higher levels (**p < 0.01) of rheumatoid factor (RF), C-reactive protein (CRP), erythrocyte sedimentation rate (ESR) at 1 h and 2 h, antinuclear antibody (ANA), and anti-cyclic citrullinated peptide (anti-CCP) antibodies in RA cases compared to controls. These parameters indicate increased inflammation and autoimmune activity in RA.

**Fig. 2:**
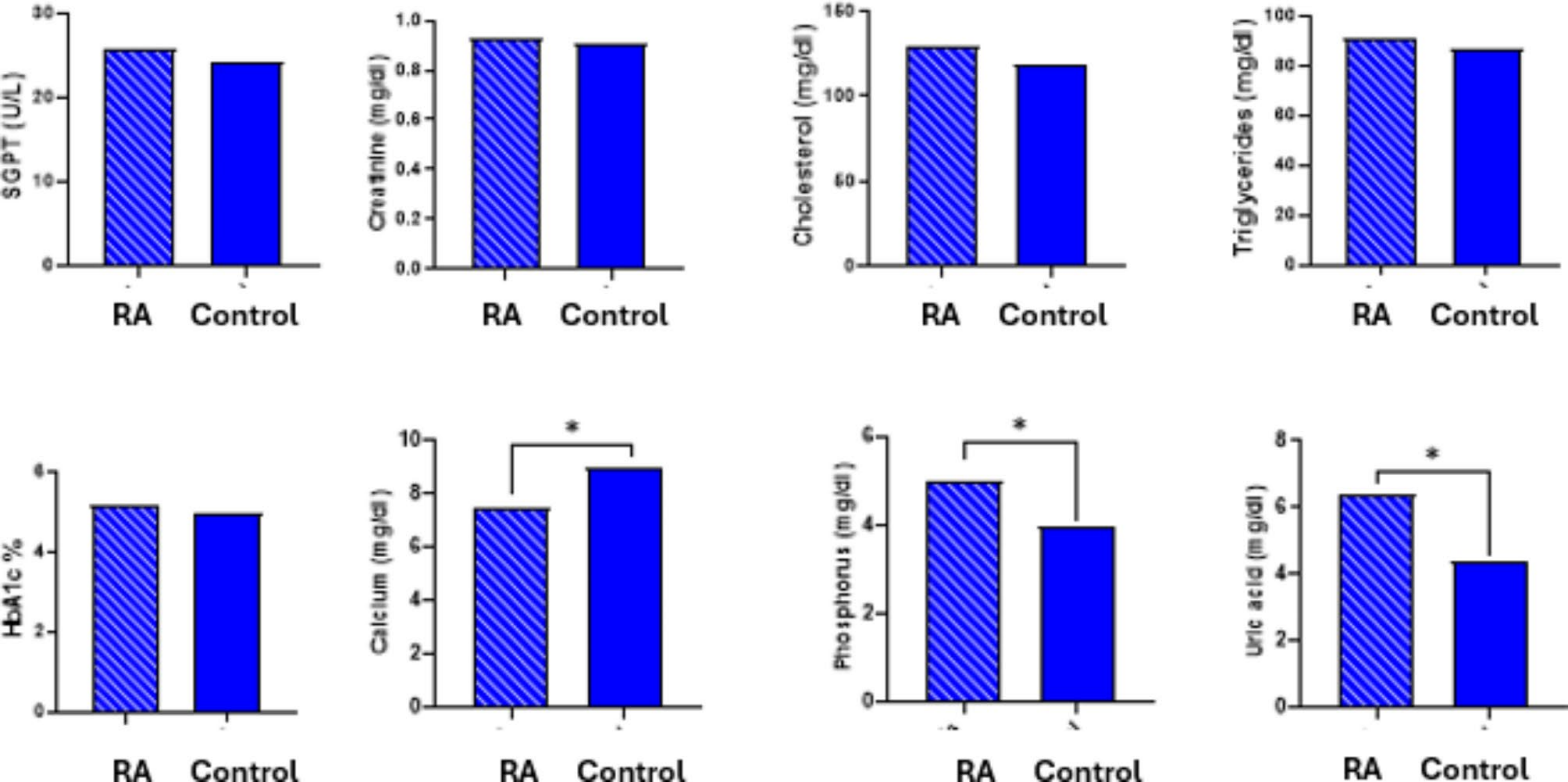
Biochemical parameters for participants with RA compared to controls. The charts show no significant differences in SGPT, creatinine, cholesterol, triglycerides, and HbA1c levels. However, significant differences (*p < 0.05) are observed in calcium, phosphorus, and uric acid levels, with lower calcium and phosphorus and higher uric acid in RA cases, indicating altered metabolic profiles in these patients.

By comparing serological parameters between control group and RA cases the analysis showed that there is a significant statistical difference in the values of RF, CRP, ANA & Anti-CCP. By comparing hematological parameters between control group and RA cases, the analysis showed that there was a significant statistical difference in the values of Hb, RBCs & ESR (1 h & 2 h). And there was no significant statistical difference in the values of WBCs and platelets. By comparing biochemical parameters between control group and RA cases, the analysis showed that there was a significant statistical difference in the values of calcium, phosphorus & uric acid. P-value for these parameters is less than 0.05 (Table 2; Figs. 1 and 2).

**Table 2:** Immunological, hematological, and biochemical parameters for all participants.

Parameters	RA *N* = 60	Control *N* = 20	t-test *P*-value
	Mean (µ ± SD)		
Immunological & Serological parameters			
RF (IU/ml)	41.17 ± 14.96	2.88 ± 1.27	0.0001
CRP (mg/L)	30.32 ± 15.56	3.66 ± 1.083	0.014
ANA (IU/ml)	1.05 ± 0.451	0.346 ± 0.141	0.036
Anti-CCP (U/ml)	147.678 ± 40.78	4.055 ± 1.623	0.009
Hematological parameters			
Hb (g/dl)	11.44 ± 1.29	12.75 ± 1.63	0.0001
RBCs (cells/ul)	4.51 ± 0.446	4.88 ± 0.345	0.001
WBCs (cells/mm)	6.59 ± 2.432	7.385 ± 2.29	0.204
Platelets (mm)	290.91 ± 82.72	282.8 ± 82.21	0.705
ESR 1 h (mm/h)	41.66 ± 23.53	5.05 ± 1.23	0.0001
ESR 2 h (mm/h)	73.5 ± 30.49	12.35 ± 2.059	0.0001
Biochemical parameters			
Calcium (mg/dl)	7.54 ± 1.65	9.09 ± 0.33	0.015
Phosphorus (mg/dl)	4.51 ± 0.417	4.07 ± 0.227	0.0001
Uric acid (mg/dl)	6.465 ± 0.624	4.455 ± 0.531	0.0001
SGPT (U/L)	25.56 ± 6.277	24.25 ± 5.485	0.405
Creatinine (mg/dl)	0.936 ± 0.158	0.918 ± 0.129	0.644
Cholesterol (mg/dl)	129.1 ± 29.947	118.25 ± 25.2	0.149
Triglycerides (mg/dl)	91.116 ± 19.165	87 ± 11.86	0.370
HbA1c %	5.291 ± 0.577	5.04 ± 0.428	0.078

### The Pearson correlation

The data reveal a robust positive correlation between rheumatoid factor (RF) and C-reactive protein (CRP) with a significant p-value of 0.0001. Additionally, there is a notable positive correlation between RF and both the erythrocyte sedimentation rate at 1 h (ESR 1 h) and anti-cyclic citrullinated peptide (ANTI-CCP), with p-values of 0.026 and 0.011, respectively. CRP also shows a significant positive correlation with ESR 1 h, ESR 2 h, and ANTI-CCP, having p-values of 0.010, 0.027, and 0.010, respectively. A strong positive correlation is also observed between ESR 1 h and ESR 2 h, with a p-value of 0.0001. Conversely, there is a negative correlation between RF and antinuclear antibody (ANA) with a p-value of 0.047. Furthermore, the Disease Activity Score (DAS) number demonstrates a strong positive correlation with RF, CRP, ESR 1 h, and ESR 2 h, with significant p-values of 0.0001, 0.015, 0.001, and 0.0001, respectively.

**Fig. 3:**
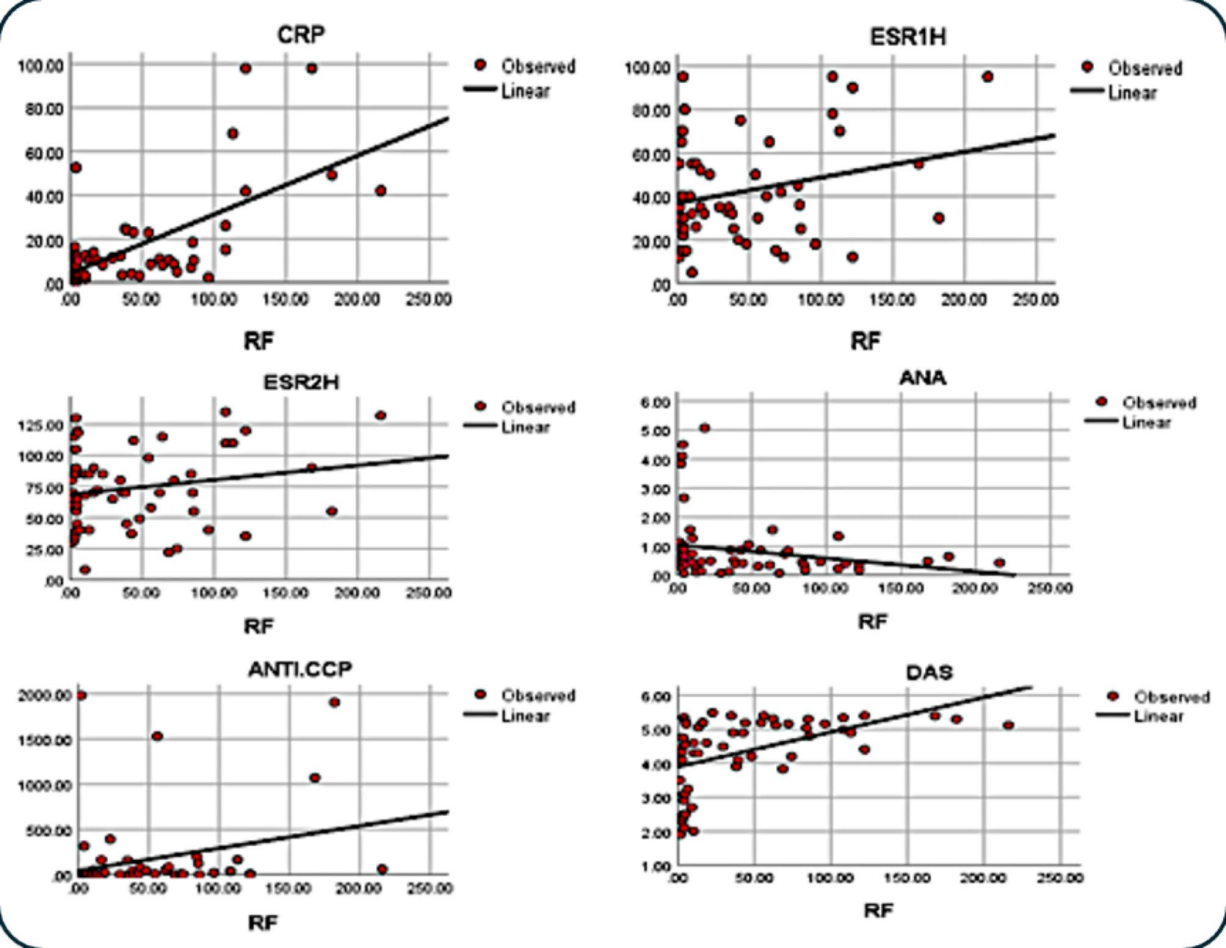
Correlation coefficients between various immunological and inflammatory parameters in RA patients. The scatter plots illustrate the relationships between rheumatoid factor (RF) and C-reactive protein (CRP), erythrocyte sedimentation rate at 1 h (ESR1h), erythrocyte sedimentation rate at 2 h (ESR2h), antinuclear antibody (ANA), anti-cyclic citrullinated peptide (ANTI-CCP), and Disease Activity Score (DAS). Strong positive correlations are observed between RF and CRP, RF and ESR1h, RF and ANTI-CCP, CRP and ESR1h, CRP and ESR2h, CRP and ANTI-CCP, ESR1h and ESR2h, and DAS with RF, CRP, ESR1h, and ESR2h. A negative correlation is noted between RF and ANA. These correlations emphasize the interconnected nature of these markers in reflecting disease activity and inflammation in RA.

These correlations highlight the interconnected nature of these immunological and inflammatory markers in RA. The strong positive correlations suggest that as the levels of RF and CRP increase, there is a concurrent rise in ESR and ANTI-CCP levels, reflecting heightened inflammatory and autoimmune activity. The negative correlation between RF and ANA may indicate different pathological roles or regulatory mechanisms in the disease process. The strong association between the DAS number and these markers further emphasizes their relevance in assessing disease activity and severity in RA patients (Table 3& Fig. 3).

### Subgroup analysis based on treatment type

A comparative analysis of RA patients treated with conventional Disease-Modifying Antirheumatic Drugs (DMARDs) and those treated with biological DMARDs revealed significant differences in the values of rheumatoid factor (RF) and Disease Activity Score (DAS).

**Table 3:** Correlations coefficient for RA cases.

		RF	CRP	ESR 1 h	ESR 2 h	ANA	ANTI-CCP
RF	**Pear. Corr.**	1	0.654^**^	0.251^*^	0.191	-0.218^*^	0.297^*^
	**p-value**		0.0001	0.026	0.072	0.047	0.011
CRP	**Pear. Corr.**	0.654^**^	1	0.298^*^	0.249^*^	-0.028	0.299^*^
	**p-value**	0.0001		0.010	0.027	0.417	0.010
ESR 1 h	**Pear. Corr.**	0.251^*^	0.298^*^	1	0.959^**^	0.042	-0.028
	**p-value**	0.026	0.010		0.0001	0.376	0.416
ESR 2 h	**Pear. Corr.**	0.191	0.249^*^	0.959^**^	1	0.080	-0.032
	**p-value**	0.072	0.027	0.0001		0.271	0.405
ANA	**Pear. Corr.**	-0.218^*^	-0.028	0.042	0.080	1	0.018
	**p-value**	0.047	0.417	0.376	0.271		0.446
ANTI-CCP	**Pear. Corr.**	0.297^*^	0.299^*^	-0.028	-0.032	0.018	1
	**p-value**	0.011	0.010	0.416	0.405	0.446	
DAS	**Pear. Corr.**	0.474**	0.311*	0.425**	0.458**	-0.041	0.234
	**p-value** **lue**	0.0001	0.015	0.001	0.0001	0.756	0.072

Pear. Corr.: Pearson Correlation, * denotes Correlation is significant at the 0.05 level, ** denotes Correlation is significant at the 0.01 level.

The analysis demonstrated that patients receiving biological DMARDs had markedly different RF and DAS values compared to those on conventional DMARDs, with p-values for these parameters being less than 0.05. This suggests that biological DMARDs may have a more pronounced effect on reducing disease activity and altering immunological markers, highlighting their potential efficacy in managing RA compared to conventional treatments. These findings underscore the importance of treatment selection in achieving optimal clinical outcomes for RA patients (Table 4).

**Table 4:** Difference between serological parameters according to type of treatment.

Parameters	Conventional DMARDs treatment (*N* = 33)	Biological DMARDs treatment (*N* = 27)	t-test *p*-value
	Mean (µ ± SD)		
RF	23.47 ± 10.92	62.1 ± 11.63	0.002
CRP	20.705 ± 13.103	20.46 ± 18.029	0.360
ESR 1 h	42.454 ± 10.756	40.33 ± 23.59	0.731
ESR 2 h	75.24 ± 31.69	71.37 ± 29.417	0.629
ANA	1.933 ± 1.23	0.640 ± 0.363	0.153
Anti-CCP	345.117 ± 99.37	487.14 ± 192.2	0.392
DAS	4.00 ± 1.115	4.70 ± 0.845	0.009

### Subgroup analysis based on duration of disease

The analysis of RA patients categorized by disease duration into three subgroups—those with a disease duration of less than 5 years, those with a duration between 5 and 10 years, and those with a duration of more than 10 years—revealed no statistically significant differences in the evaluated parameters. The p-values for these parameters were all greater than 0.05, indicating that the duration of the disease does not significantly impact the measured immunological and inflammatory markers. This finding suggests that, regardless of whether patients have had RA for a short term, medium term, or long term, the levels of markers such as RF, CRP, ESR, ANA, and ANTI-CCP, as well as the DAS number, remain comparable across these groups. This underscores the chronic and persistent nature of the disease, where disease duration alone may not be a determinant of marker variation or disease activity (Table 5).

**Table 5:** Difference between parameters according to disease duration.

Parameters	Duration (1–5 y) *N* = 30	Duration (5–10 y) *N* = 15	Duration > 10 y *N* = 15	F-TEST *p*-value
	Mean (µ ± SD)			
RF	31.973 ± 9.053	49.374 ± 12.748	52.068 ± 13.44	0.379
CRP	13.99 ± 10.58	19.19 ± 12.182	14.11 ± 10.227	0.708
ESR 1 h	47.8 ± 24.85	37 ± 20.77	33.266 ± 20.892	0.099
ESR 2 h	81.60 ± 30.706	68.66 ± 28.434	62.13 ± 29.218	0.100
ANA	1.85 ± 0.986	1.98 ± 1.31	1.746 ± 0.964	0.836
Anti-CCP	360.48 ± 103.96	597.13 ± 261.94	274.67 ± 94.72	0.433
DAS	4.26 ± 1.047	4.56 ± 0.834	4.19 ± 1.278	0.577

### Parameters of RA according to family history

An analysis comparing RA (RA) parameters between patients with a positive family history of the disease and those without revealed no significant statistical differences. The parameters assessed included various immunological and inflammatory markers such as rheumatoid factor (RF), C-reactive protein (CRP), erythrocyte sedimentation rate (ESR), antinuclear antibody (ANA), anti-cyclic citrullinated peptide (ANTI-CCP), and Disease Activity Score (DAS). The p-values for these comparisons were all greater than 0.05, indicating that having a family history of RA does not significantly affect the levels of these markers or the overall disease activity (Table 6).

**Table 6:** Difference between parameters regarding family history of RA.

Parameters	Family history of RA (*N* = 31)	No family history of RA (*N* = 29)	t-test *p*-value
	Mean (µ ± SD)		
RF	42.47 ± 23.83	39.13 ± 26.86	0.799
CRP	14.14 ± 10.56	16.57 ± 11.85	0.652
ESR 1 h	37.709 ± 20.89	45.55 ± 25.76	0.199
ESR 2 h	69.80 ± 28.57	77.44 ± 32.45	0.336
ANA	1.874 ± 1.14	1.83 ± 0.974	0.895
Anti-CCP	325.83 ± 108.78	494.87 ± 175.73	0.536
DAS	4.12 ± 1.21	4.53 ± 0.82	0.131

### Parameters according to DAS Number subgroups

An analysis of RA (RA) patients categorized by Disease Activity Score (DAS) subgroups revealed significant statistical differences in the values of several key parameters. Specifically, the levels of rheumatoid factor (RF), erythrocyte sedimentation rate at 1 h (ESR1h), erythrocyte sedimentation rate at 2 h (ESR2h), and the DAS itself showed marked variations across different DAS subgroups. The p-values for these parameters were all less than 0.05, indicating statistically significant differences (Table 7).

**Table 7:** Comparison between parameters according to DAS number subgroups.

Parameters	DAS number (Remission)	DAS number (Low)	DAS number (Moderate)	DAS number (High)	*P*-value
	Mean (µ ± SD)				
RF	4.3 ± 2.58	5.2 ± 2.51	35.07 ± 30.37	70.72 ± 62.4	0.002
CRP	7.07 ± 5.961	2.27 ± 1.645	14.07 ± 21.02	22.97 ± 20.32	0.121
ESR 1 h	29.5 ± 24.34	30.0 ± 12.24	36.25 ± 19.40	55.95 ± 24.28	0.005
ESR 2 h	52.37 ± 35.18	67.5 ± 25.33	67.071 ± 26.42	92.15 ± 26.756	0.003
ANA	0.625 ± 0.301	1.65 ± 1.22	1.917 ± 1.141	1.705 ± 0.957	0.375
Anti-CCP	9.25 ± 4.272	14.57 ± 11.32	372.51 ± 95.3	547.51 ± 283.36	0.278
DAS	2.27 ± 0.243	2.94± 0.186	4.42 ± 0.449	5.27 ± 0.11	0.0001

### ROC curve analysis for RA and control

**Fig. 4:**
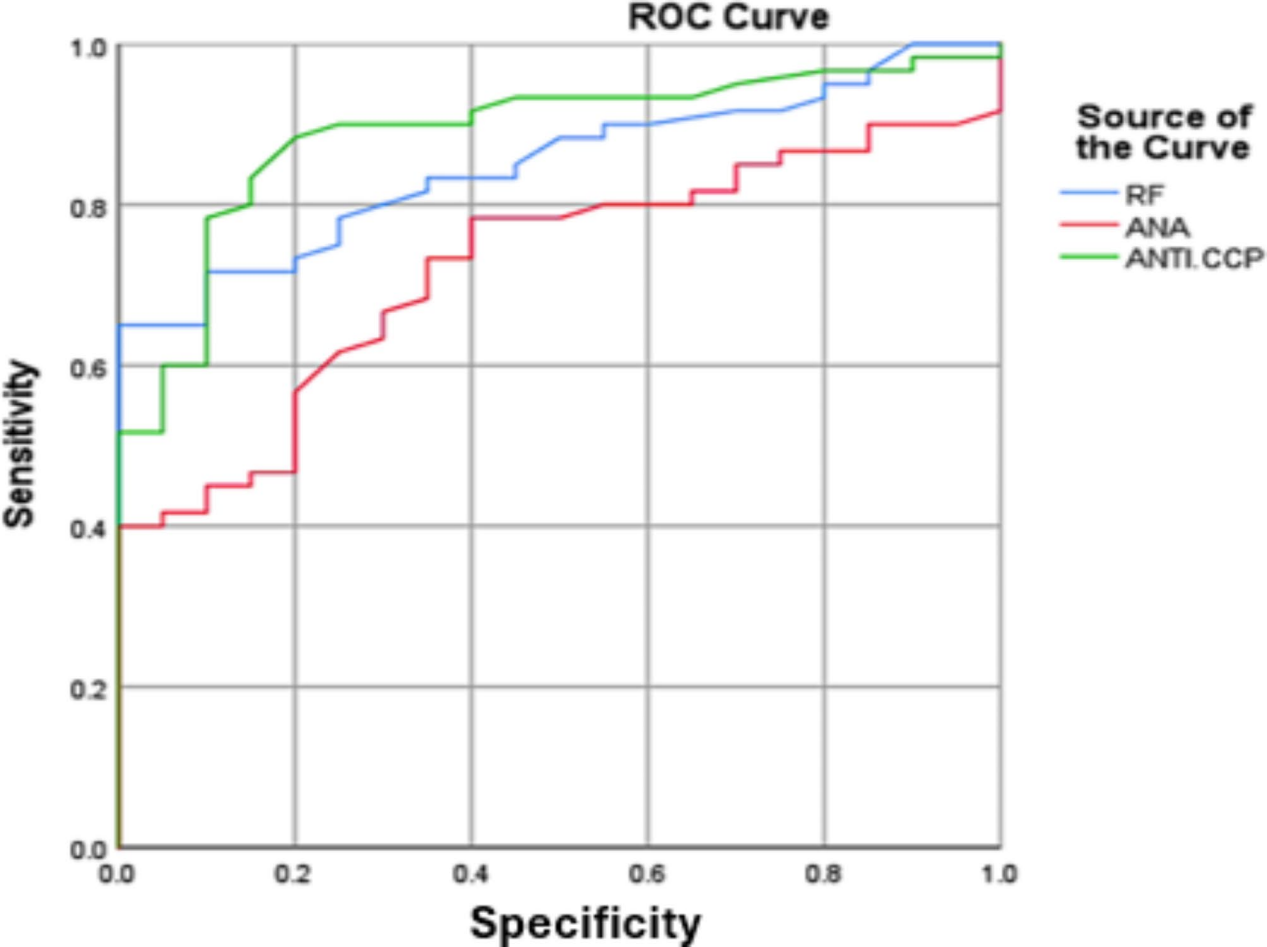
ROC curves comparing the diagnostic performance of rheumatoid factor (RF), antinuclear antibody (ANA), and anti-cyclic citrullinated peptide (ANTI-CCP) for RA. The ANTI-CCP test (green line) exhibits the highest AUC of 0.965 with a sensitivity of 78% and specificity of 90%, followed by the RF test (blue line) with an AUC of 0.849, sensitivity of 65%, and specificity of 100%. The ANA test (red line) shows an AUC of 0.721 with a sensitivity of 40% and specificity of 100%. These curves indicate that ANTI-CCP is the most effective marker for RA diagnosis, followed by RF and ANA.

The Receiver Operating Characteristic (ROC) curve analysis for differentiating RA (RA) patients from control subjects demonstrated that the anti-cyclic citrullinated peptide (Anti-CCP) test exhibited the highest diagnostic performance. The Anti-CCP test had an area under the curve (AUC) of 0.965, with a sensitivity of 78% and a specificity of 90% (p-value < 0.0001), indicating its superior accuracy in identifying RA. In comparison, the antinuclear antibody (ANA) test showed an AUC of 0.721, with a sensitivity of 40% and a specificity of 100% (p-value = 0.003). Although ANA has a lower sensitivity, its perfect specificity indicates that it is highly reliable in confirming RA when the test result is positive. The rheumatoid factor (RF) test also demonstrated strong diagnostic capability with an AUC of 0.849, sensitivity of 65%, and specificity of 100% (p-value < 0.0001). This suggests that while RF is a useful marker for RA, it is less sensitive than Anti-CCP but equally specific. Overall, the ROC curve analysis underscores the diagnostic value of these tests, with Anti-CCP being the most effective for early and accurate RA detection, followed by RF, which is highly specific, and ANA, which is particularly valuable for confirming RA due to its high specificity despite lower sensitivity. These findings highlight the importance of using a combination of these markers to improve diagnostic accuracy in clinical practice (Table 8; Fig. 4).

**Table 8:** ROC curve analysis for parameters of RA cases vs. control.

Parameters	AUC	Cut-off Value	Sensitivity %	Specificity %	*p*-value	95% CI	
						Lower Bound	Upper Bound
RF	0.849	5.25	65%	100%	0.0001	0.767	0.931
ANA	0.721	0.625	40%	100%	0.003	0.609	0.833
ANTI-CCP	0.889	5.45	78%	90%	0.0001	0.813	0.965

### miRNAs analysis

**Fig. 5:**
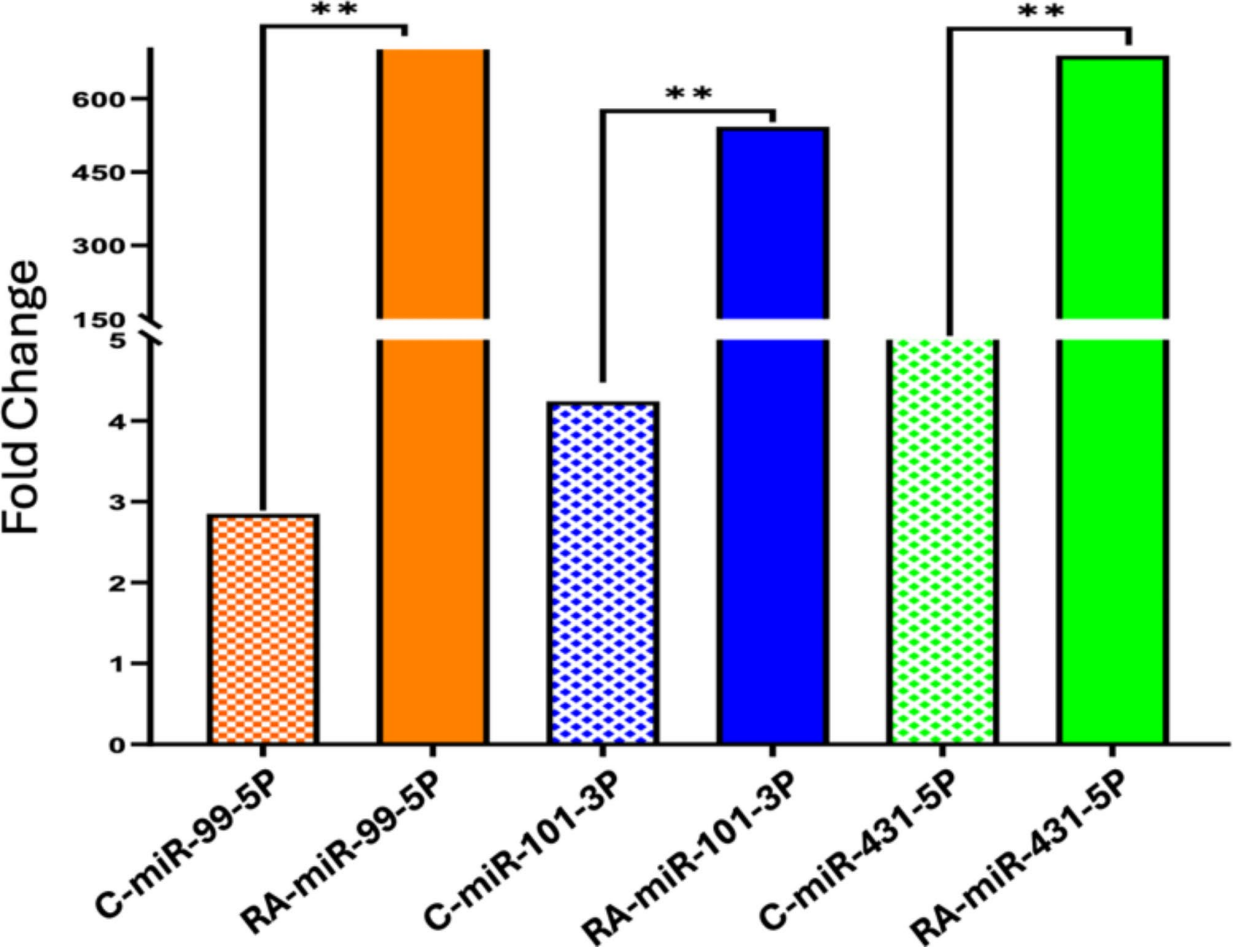
Fold change in the expression levels of miR-99b-5p, miR-101-3p, and miR-431-5p in RA patients compared to healthy controls. This bar graph illustrates the relative expression levels of three different miRNAs (miR-99b-5p, miR-101-3p, and miR-431-5p) between RA patients and healthy controls. The y-axis represents the fold change in expression, indicating how much the expression levels of these miRNAs are increased or decreased in RA patients compared to the control group.

In the present study, the expression levels of three specific microRNAs (miRNAs) were evaluated in patients with RA and compared to a healthy control group using quantitative Polymerase Chain Reaction (qPCR). The miRNAs examined were miR-99b-5p, miR-101-3p, and miR-431-5p. These miRNAs are known to play significant roles in regulating gene expression and can act as biomarkers for various diseases, including RA. Specifically, the p-values for miR-99b-5p, miR-101-3p, and miR-431-5p were 0.012, 0.004, and 0.0001, respectively (Table 9). These p-values indicate that the differences in expression levels are statistically significant, suggesting a strong association with RA (Fig. 5).

**Table 9:** The mean FC of different miRNAs for RA and control groups.

Parameters	Rheumatoid arthritis cases *N* = 60	Control *N* = 20	*P*-value
	Mean (µ ± SD)		
miRNA 99b-5p	808.544 ± 273.48	2.850 ± 1.745	0.012
miRNA 101-3p	542.087 ± 147.708	4.24 ± 2.007	0.004
miRNA 431-5p	687.931 ± 434.448	10.898 ± 8.232	0.0001

### Circulating miRNAs for treatment categories subgroups

**Fig. 6:**
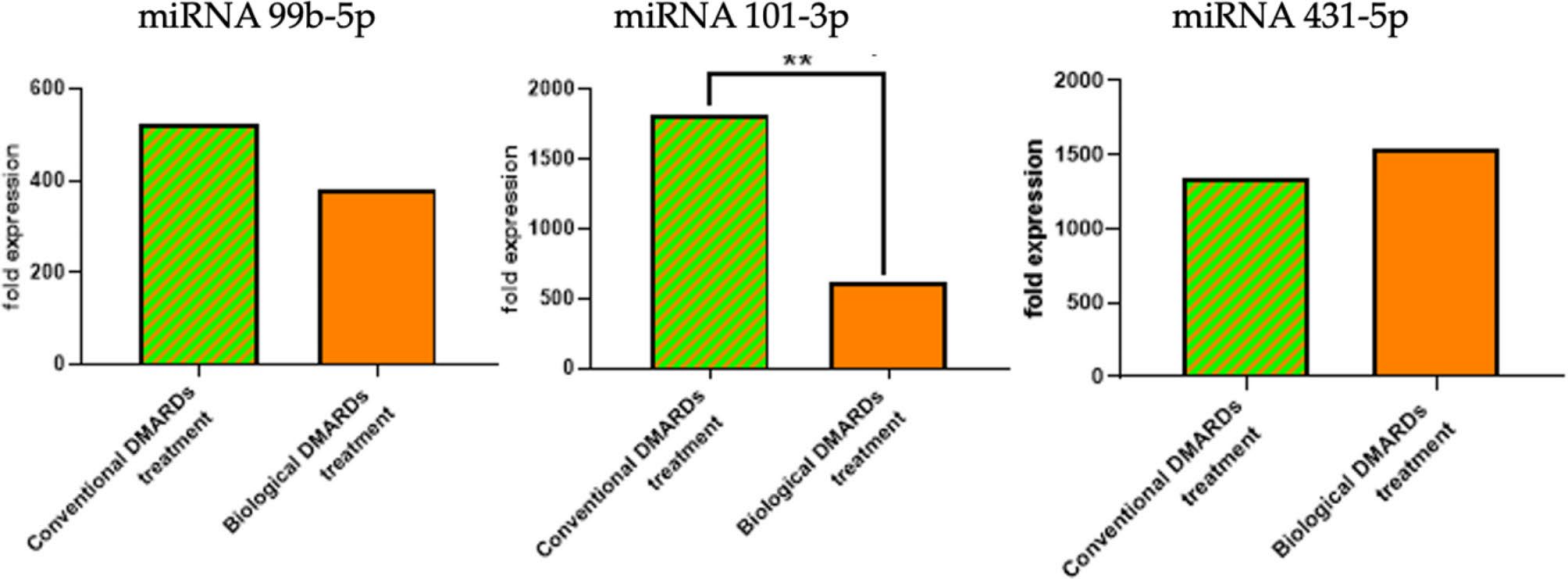
Comparison of fold expression levels of miR-99b-5p, miR-101-3p, and miR-431-5p in RA patients undergoing conventional DMARDs treatment versus biological DMARDs treatment. miR-99b-5p (Left): Shows similar expression levels between the two treatment groups. miR-101-3p (Middle): Displays a significantly higher expression in patients treated with conventional DMARDs compared to those treated with biological DMARDs, indicated by the double asterisks (**), highlighting a significant statistical difference. miR-431-5p (Right)**: Indicates higher expression levels in the biological DMARDs treatment group compared to the conventional DMARDs group.

In the present study, the expression levels of circulating miRNAs were analyzed across different treatment categories for RA patients. The analysis involved comparing the mean ± standard deviation (SD) of miRNA expression levels among these subgroups. The results revealed significant statistical differences, particularly in the expression level of miR-101-3p. Specifically, the p-value for miR-101-3p was found to be 0.030, indicating a meaningful variation in its expression depending on the treatment category (Fig. 6).

### Circulating miRNAs according to disease duration

**Fig. 7:**
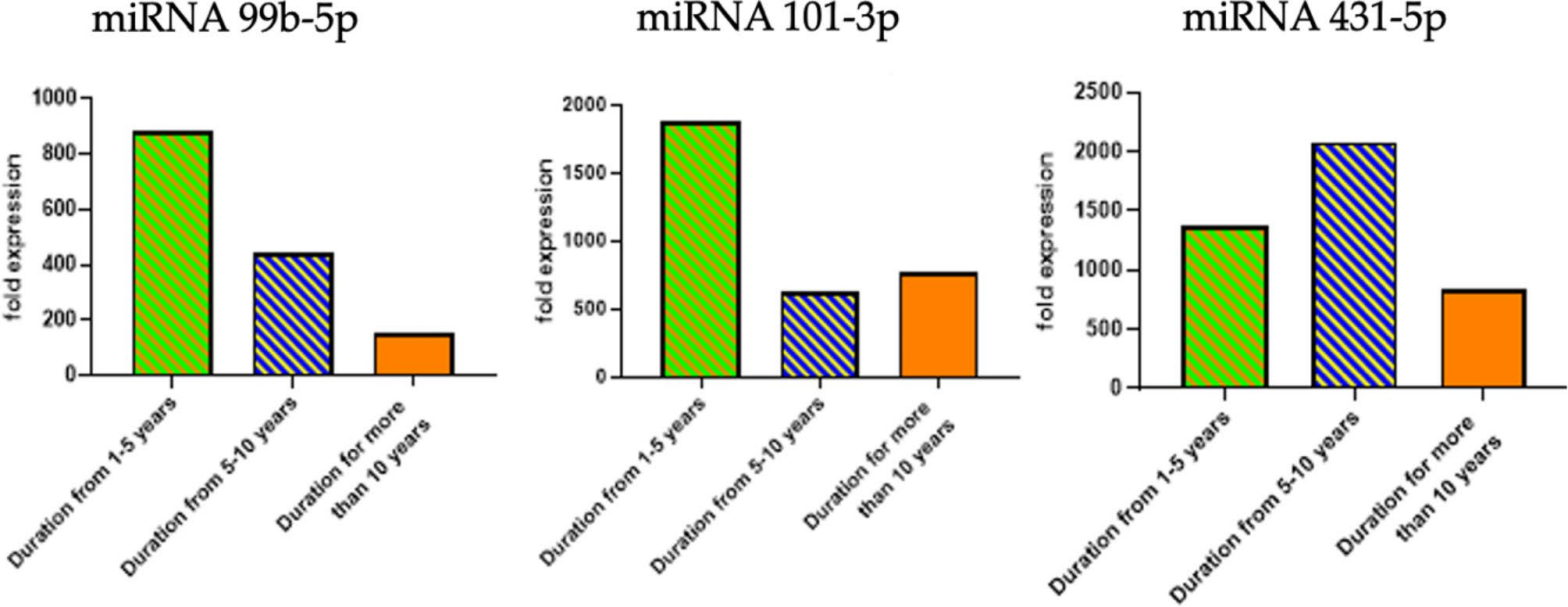
Expression levels of circulating miRNAs (miR-99b-5p, miR-101-3p, and miR-431-5p) in RA patients categorized by disease duration. miR-99b-5p (Left): Expression is highest in patients with disease duration of 1–5 years, decreasing significantly in those with 5–10 years, and even more in those with over 10 years. miR-101-3p (Middle): Shows a similar trend, with the highest expression in the 1–5 years group and decreasing levels in the subsequent groups. miR-431-5p (Right): Exhibits higher expression in the 1-5- and 5-10-years groups compared to those with more than 10 years.

In this study, the expression levels of circulating miRNAs (miR-99b-5p, miR-101-3p, and miR-431-5p) were analyzed in RA patients categorized by the duration of their disease. The analysis compared the mean ± standard deviation (SD) of miRNA expression levels across different duration subgroups to determine if the length of time a patient had the disease impacted these levels. The expression levels of miR-99b-5p, miR-101-3p, and miR-431-5p did not show significant differences across the duration subgroups. These findings suggest that the duration of RA does not significantly influence the expression levels of the studied miRNAs (Fig. 7).

### Circulating miRNAs and family history

**Fig. 8:**
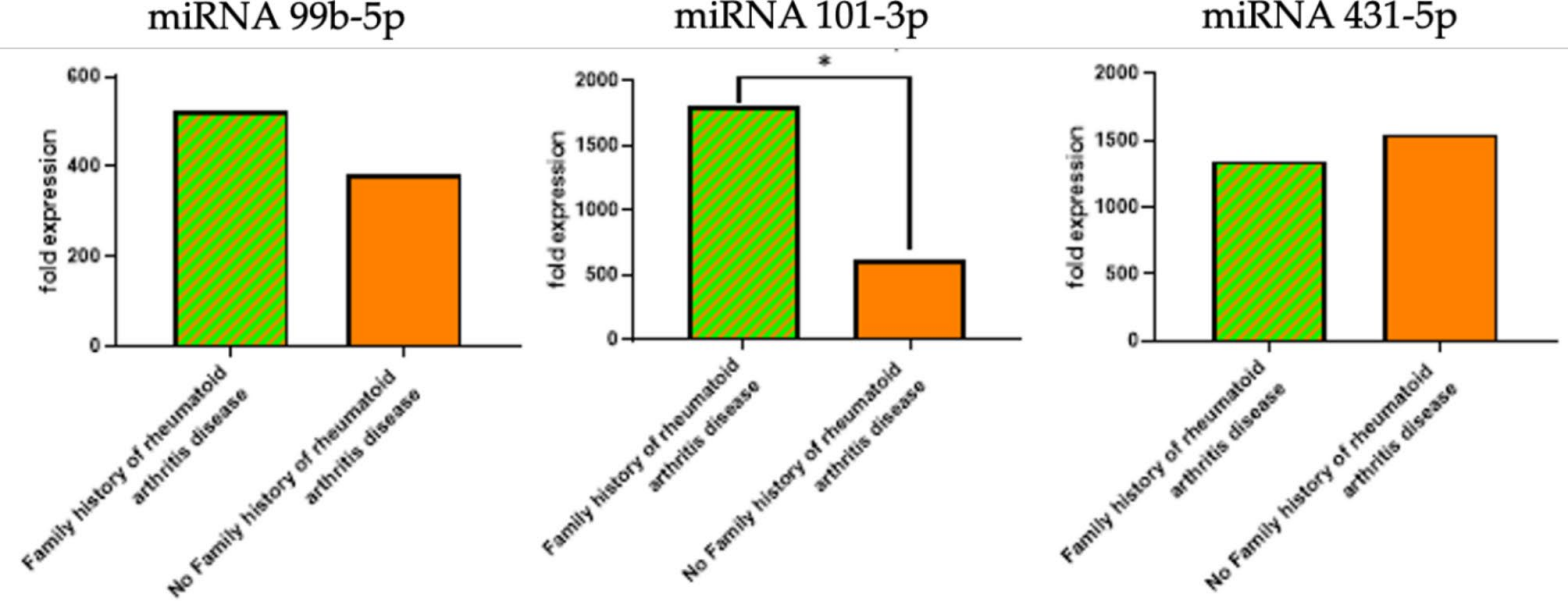
Expression levels of circulating miRNAs (miR-99b-5p, miR-101-3p, and miR-431-5p) in RA patients with and without a family history of the disease. The bar graphs depict the fold expression levels of three miRNAs in RA patients, comparing those with a family history of the disease to those without. miR-99b-5p (Left): Shows similar expression levels between patients with and without a family history of RA. miR-101-3p (Middle): Displays significantly higher expression in patients with a family history of the disease compared to those without, indicated by the asterisk (*), highlighting a significant statistical difference. miR-431-5p (Right): Indicates no significant difference in expression levels between the two groups.

The expression levels of circulating miRNAs were analyzed in RA patients with and without a family history of the disease. The aim was to determine if there were any significant differences in miRNA expression levels based on family history. For miR-99b-5p and miR-431-5p, no significant statistical differences were observed in the expression levels between the two subgroups, while miR-101-3p revealed a significant statistical difference in its expression levels between the two subgroups (Fig. 8).

### Circulating miRNAs and DAS number categories

**Fig. 9:**
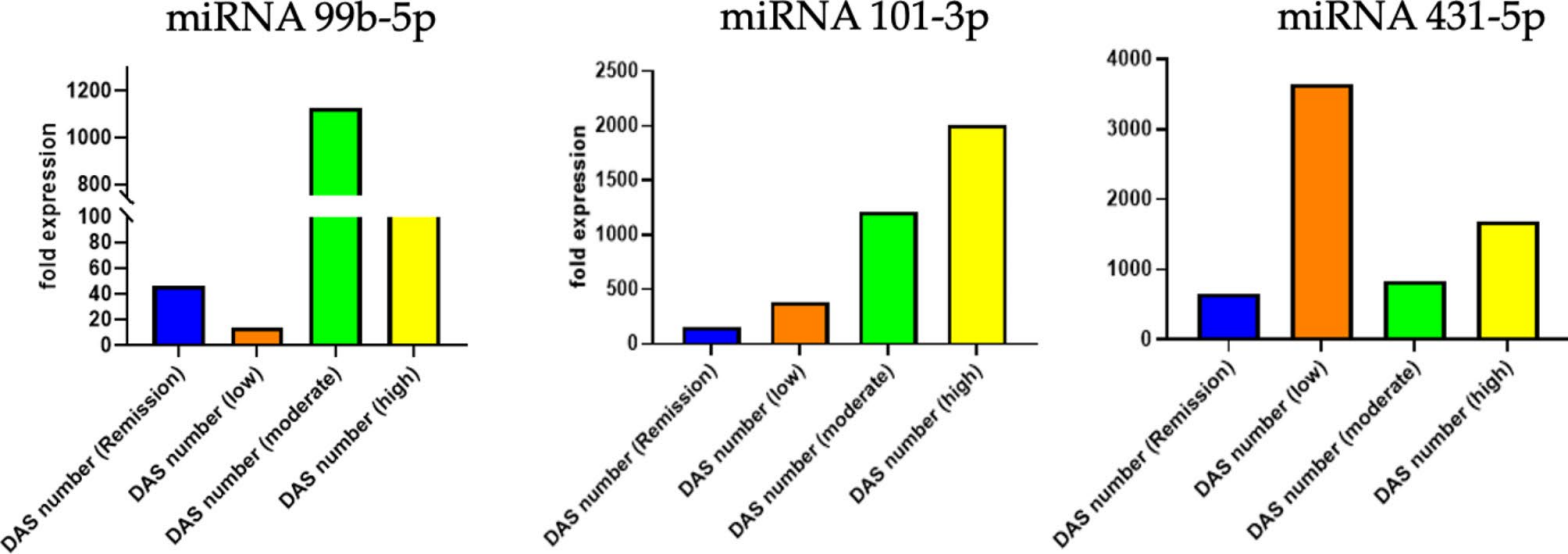
Expression levels of circulating miRNAs (miR-99b-5p, miR-101-3p, and miR-431-5p) in RA patients categorized by Disease Activity Score (DAS) into remission, low, moderate, and high disease activity. miR-99b-5p (Left): Shows variable expression with higher levels in moderate and high DAS categories. miRNA-101-3p (Middle): Indicates increased expression in patients with high disease activity. miR-431-5p (Right): Exhibits elevated levels in the low DAS category compared to remission and moderate categories.

The expression levels of circulating miRNAs were analyzed in RA patients categorized by their Disease Activity Score (DAS). The DAS is a measure used to assess the severity and activity of RA in patients. Data indicated that no significant differences in expression levels of the three miRNAs studied among patients with different DAS scores (Fig. 9).

### Correlation coefficient between serological parameters and the expression level of circulating miRNAs

**Fig. 10:**
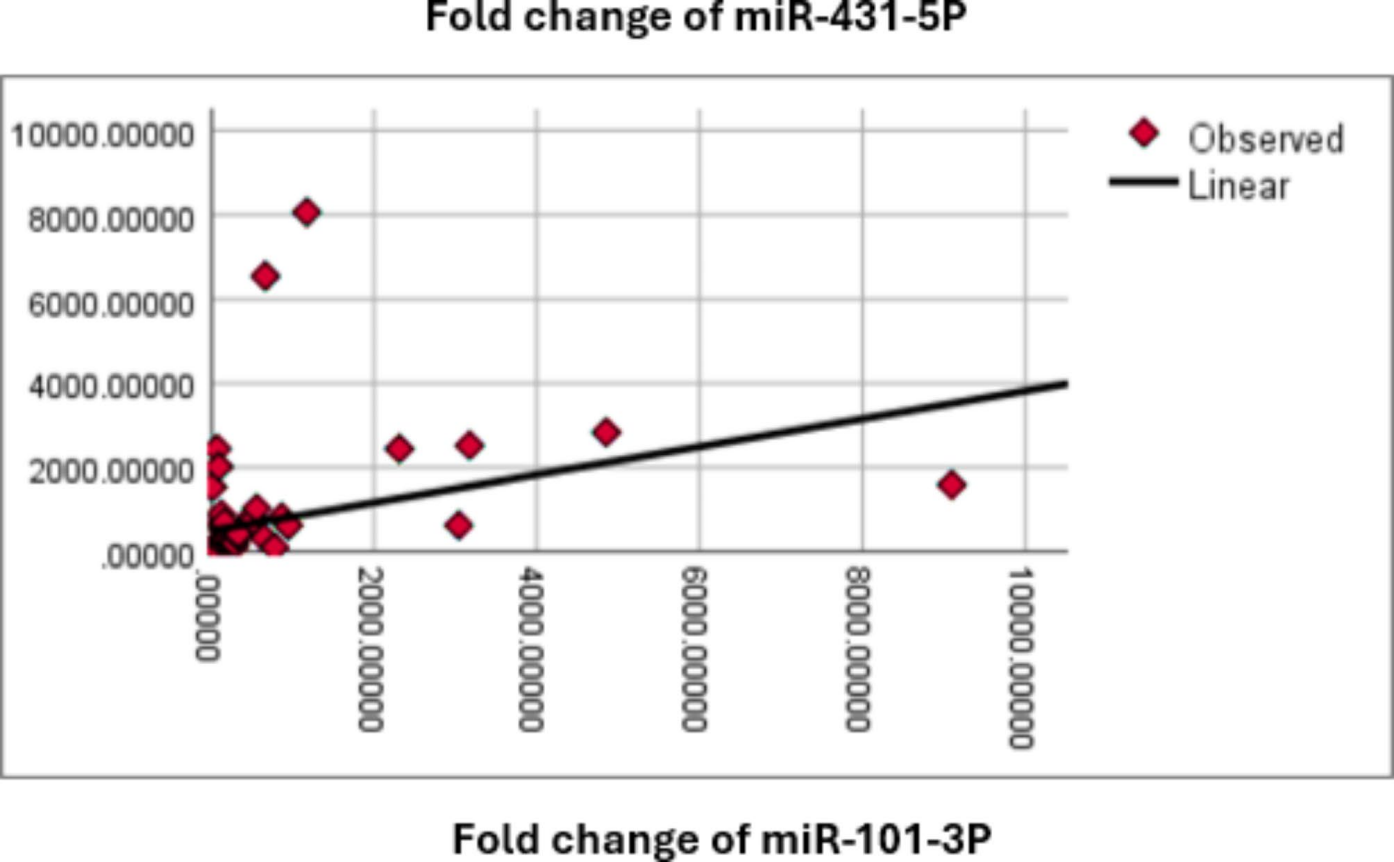
Scatter plot with a linear regression line for the correlation between expression level of miR-101-3p and miR-431-5p. The plot demonstrates a positive correlation between the variables, as indicated by the upward slope of the regression line.

In the context of RA, the study examined the correlation between serological parameters and the expression levels of circulating miRNAs. The Pearson correlation coefficient was utilized to measure the strength and direction of the linear relationship between these variables. The positive correlation between the expression levels of miR-101-3p and miR-431-5p in RA patients highlights a potential link in their regulatory roles in the disease. Understanding these correlations can help in identifying miRNA networks involved in RA pathogenesis and might offer new avenues for therapeutic targets and biomarkers for disease monitoring. Further research is needed to explore these relationships in detail and to understand the underlying mechanisms driving these correlations (Tables 10and Fig. 10).

**Table 10:** The correlation coefficient between mean values (µ ± SD) of serological parameters and the expression level of circulating miRNAs for RA cases.

		miR-99b-5p	miR-101-3p	miR-431-5p	RF	CRP	ESR (1 h)	ESR (2 h)	ANA	ANTI-CCP	DAS
miR-99b-5p	Pear. Corr.	1	-0.0015	-0.058	-0.004	-0.069	-0.074	-0.124	-0.113	-0.104	0.007
	p-value		0.912	0.661	0.977	0.602	0.576	0.345	0.389	0.431	0.956
miR-101-3p	Pear Corr.	-0.015	1	0.328^*^	0.010	0.205	0.041	0.045	-0.151	-0.100	0.080
	p-value	0.912		0.011	0.938	0.117	0.756	0.734	0.250	0.449	0.544
miR-431-5p	Pear. Corr.	-0.058	0.328^*^	1	-0.052	0.029	-0.091	− 0.008	-0.189	-0.134	-0.060
	p-value	0.661	0.011		0.693	0.828	0.489	0.951	0.149	0.308	0.651

Pear. Corr.: Pearson Correlation, * denotes Correlation is significant at the 0.05 level.

### ROC curve analysis for miRNAs

**Fig. 11:**
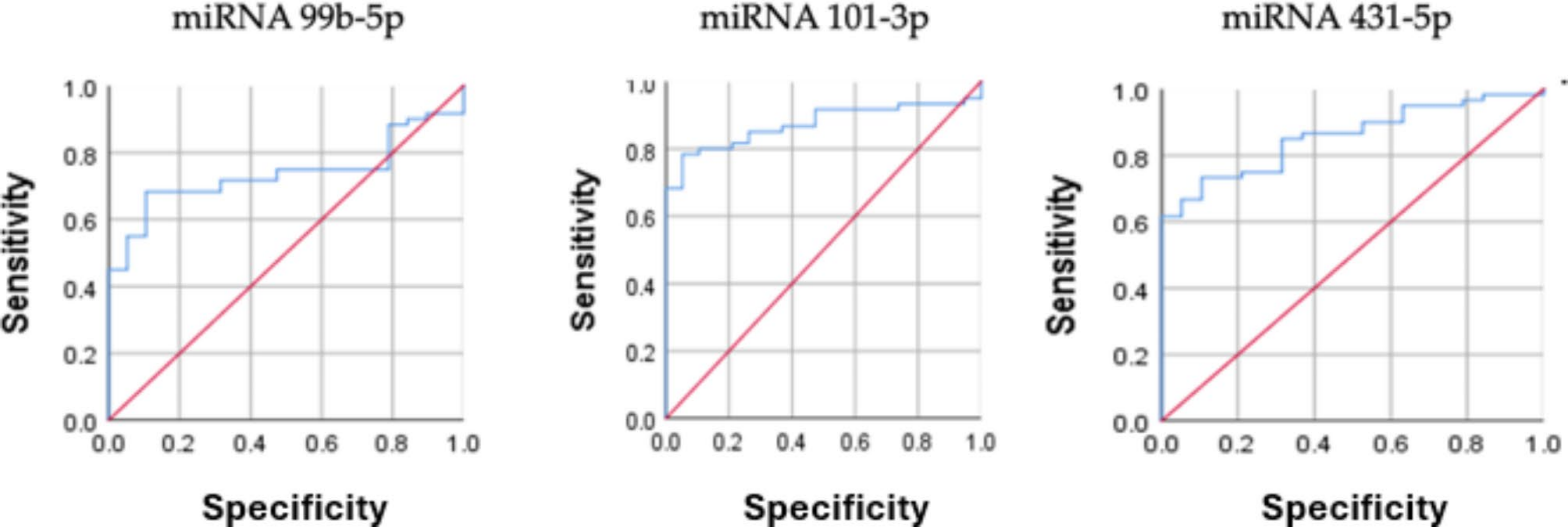
ROC Curve Analysis for miRNAs in RA Diagnosis. The ROC curves illustrate the diagnostic performance of three miRNAs in distinguishing RA among participants. The first curve represents miR-101-3p, showing the highest Area Under the Curve (AUC) of 0.873, with a sensitivity of 78% and specificity of 94% (p-value < 0.0001). The second curve corresponds to miR-431-5p, with an AUC of 0.856, sensitivity of 73%, and specificity of 89% (p-value = 0.003). The third curve depicts miR-99b-5p, with an AUC of 0.737, sensitivity of 68%, and specificity of 89% (p-value < 0.0001). These curves demonstrate the potential of these miRNAs as reliable biomarkers for the early detection of RA.

The ROC (Receiver Operating Characteristic) curve analysis was conducted to evaluate the diagnostic performance of various miRNAs for detecting RA among participants. The analysis revealed that miR-101-3p exhibited the highest diagnostic accuracy, with an Area Under the Curve (AUC) of 0.873. This miRNA demonstrated a sensitivity of 78% and a specificity of 94%, indicating its strong potential as a biomarker for RA (p-value = 0.000). In comparison, miR-431-5p also showed high diagnostic accuracy with an AUC of 0.856. It achieved a sensitivity of 73% and a specificity of 89%, further supporting its role as a useful biomarker in identifying RA (p-value = 0.003). Additionally, miR-99b-5p was found to have an AUC of 0.737. Although its sensitivity was lower at 68%, it maintained a high specificity of 89%, making it a valuable candidate for inclusion in a multi-biomarker panel for RA diagnosis (p-value < 0.0001) (Tables 11and Fig. 11).

**Table 11:** ROC curve analysis for miRNAs for RA among participants.

miRNAs	AUC	Cut-off value	Sensitivity %	Specificity %	*p*-value	95% CI	
						Lower Bound	Upper Bound
miR-99b-5p	0.737	3.2	68%	89%	0.002	0.630	0.844
miR-101-3p	0.873	4.8	78%	94%	0.0001	0.796	0.949
miR-431-5p	0.856	7.1	73%	89%	0.0001	0.775	0.937

## Discussion

Rheumatoid arthritis is a chronic inflammatory autoimmune disease that affects multiple systems in the body and has no known cause. The disease primarily targets synovial joints, leading to persistent inflammation and progressive destruction of both cartilage and bone within the joint, resulting in pain and functional impairment. Clinically, RA typically presents with symmetrical joint involvement, which can manifest as arthralgia, edema, redness, and restricted range of motion^15^.

MicroRNAs (miRNAs) are small, non-coding RNAs that regulate various biological functions. They have been linked to many diseases, including autoimmune disorders, neurological conditions, viral infections, and cancers. Recent research has identified specific genes, mutations, and miRNAs that contribute to the etiology of RA. Due to their crucial roles in cellular regulatory processes and immune responses, miRNAs are studied as potential biomarkers for diagnosing RA, targeting treatments, monitoring disease activity, and guiding therapy^16^. Analyzing miRNAs is essential for understanding the pathogenesis of RA and their potential clinical applications. Integrating miRNA profiling into clinical practice could enhance early diagnosis and personalized treatment of RA, leading to improved patient outcomes^17,18^.

The gender effect on RA disease characteristics among a cohort of Egyptian patients revealed a female-to-male ratio of 5:1, with a mean patient age of 45.7 ± 10.8 years^19,20^. Our study found that approximately half of the patients had a positive family history of RA (52%) and had been living with RA for 1 to 5 years (50%). Regarding treatment types, the majority were treated with conventional DMARDs (55%), while 45% were treated with biological DMARDs.

These findings align with previous research indicating that individuals with a first-degree relative with RA are more than twice as likely to develop RA compared to the general population^21,22^. Kronzer, Crowson^21^ discussed the increased risk associated with familial RA, emphasizing the importance of genetic factors in the disease’s etiology. Similarly, Frisell, Saevarsdottir^22^ highlight the significant hereditary component of RA, noting the elevated risk among first-degree relatives of affected individuals.

Our results are consistent with other studies that have examined the epidemiology and familial aggregation of RA. For instance, Sparks, Chang^23^ reported a similar female predominance and an increased risk of RA among relatives, supporting the notion that genetic predisposition plays a critical role in RA susceptibility.

Moreover, the treatment patterns observed in our study reflect current therapeutic approaches for RA, where conventional DMARDs are often the first line of treatment, and biological DMARDs are reserved for more severe or refractory cases. This treatment distribution is supported by guidelines from the American College of Rheumatology and the European League Against Rheumatism, which recommend starting with conventional DMARDs and progressing to biological DMARDs as needed based on disease severity and patient response^24^. Overall, our findings contribute to the growing body of evidence highlighting the significant gender disparity, genetic predisposition, and treatment patterns in RA.

Regarding the serological parameters, our results revealed that there was a significant difference in the values of RF, CRP, ESR (1 h & 2 h), ANA, and Anti-CCP between the RA cases and the control group. These results agreed with Ghozlani, Mounach^25^ who found that RF and anti-CCP levels are linked with progression of RA and disease severity. Data also were in line with Matsuo, Tabuchi^26^ who reported that CRP and ESR levels were significantly different between RA patients and healthy controls, demonstrating continuous inflammation and disease activity in RA. Furthermore, it is stated that, since RA is an autoimmune disease, many people with RA have positive ANA tests^27^. Anti-CCP is present in 23% of patients with early-stage RA, in about 50% of patients at diagnosis, and in about 53–70% of patients 2 years after diagnosis^28^. This is consistent with our findings, which highlight the importance of these markers in diagnosing and monitoring RA.

By comparing hematological parameters between the control group and the RA group, the statistical analysis in our study showed that there is a significant difference in the values of Hb and RBCs. It has been found that RA patients’ health is impacted by anemia, a common characteristic hematological condition^29^.

Regarding the biochemical parameters, comparison between the healthy control and RA patient groups in the present study revealed a significant difference in the values of calcium, phosphorus, and uric acid, where calcium was significantly decreased while phosphorus and uric acid were significantly increased. These results were parallel to the findings of Ioannidou, Tsiakalidou^30^ who reported similar biochemical imbalances in RA patients, noting decreased calcium and increased phosphorus. Our results were also in line with Ge, Tong^31^ and Månsson, Carey^32^ who also observed these patterns, highlighting the altered mineral metabolism in RA patients.

The significant decrease in serum calcium in the patient group may be the result of an increase in calcium metabolism, an expansion of the extracellular fluid compartment, or an increase in glomerular filtration of calcium and serum phosphate, because calcium and phosphate metabolism are closely related and controlled by vitamin D^33^. Meanwhile, the significant increase in uric acid levels indicates an increase in reactive oxygen species and tissue damage in RA patients. This finding aligns with previous studies, such as Ioannidou, Tsiakalidou^30^, which established that oxidative stress plays a role in the progression of the RA condition.

Regarding microRNAs, the present study compared the expression levels of circulating miRNAs among RA cases and control cases, and it was found that circulating miR-99b-5p, miR-101-3p, and miR-431-5p significantly showed upregulation among RA patients compared to control cases. These findings are consistent with previous studies that have identified altered miRNA expression profiles in RA patients. For example, Zhu, Wu^34^ found that miRNA 99b-5p plays a role in the regulation of inflammation and immunological responses in RA, which supports its overexpression in patients.

In addition, there were significantly higher expression levels of miR-101-3p among patients treated with conventional DMARDs compared to those treated with biological DMARDs. This suggests that miR-101-3p may play a role in the therapeutic response to different DMARD treatments. A study by Bure, Mikhaylenko^35^ also reported differential miRNA expression in RA patients based on treatment types, highlighting the potential of miRNAs as biomarkers for treatment response.

The circulating miR-99b-5p and 101-3p showed higher expression levels among RA patients with disease durations of 1–5 years compared to other groups; however, this was not significant. However, the expression level of miR-101-3p was significantly higher among patients with no family history of RA disease, indicating that miRNA expression may be influenced by genetic factors and disease progression. Recent research by Gauri, Liyakat^36^ supports this finding, showing that miRNA expression can vary based on disease duration and genetic background in RA patients.

Additionally, the present results revealed a positive correlation between the expression levels of miR-101-3p and miR-431-5p. This suggests a potential co-regulatory mechanism or shared pathways involving these miRNAs in RA pathogenesis. The synergistic roles of miRNAs in regulating inflammatory pathways in autoimmune diseases was also discussed^37,38^, which aligns with our observations.

The ROC curve analysis showed that miR-101-3p expression had the greatest AUC of 0.873, with a sensitivity of 78% and a specificity of 94%. This high diagnostic performance suggests that miR-101-3p could serve as a valuable biomarker for RA diagnosis. Gong, Zhang^39^ also reported high diagnostic accuracy of specific miRNAs in distinguishing RA patients from healthy controls, further supporting our findings.

## Conclusion

In conclusion, our study’s findings highlight the potential of miRNAs as crucial biomarkers in diagnosing and managing RA. The significant upregulation of circulating miR-99b-5p, miR-101-3p, and miR-431-5p among RA patients underscores their role in the disease’s pathogenesis and their utility in clinical practice. Notably, miR-101-3p showed higher expression in patients treated with conventional DMARDs, indicating its potential in guiding treatment decisions. The positive correlation between miR-101-3p and miR-431-5p suggests shared regulatory mechanisms, which could provide insights into RA’s underlying pathways. Future studies should explore miRNA profiling to improve early diagnosis, monitor disease progression, and personalize treatment strategies, ultimately enhancing patient outcomes and paving the way for more effective RA management. Further studies involving large sample size should be conducted to evaluate the expression of other miRNAs as potential diagnostic biomarkers for RA.

## Data Availability

All data generated or analyzed during this study are included in this article.
